# Comparative effectiveness of primary tumor resection in patients with stage III pancreatic adenocarcinoma

**DOI:** 10.1186/s12885-019-5966-9

**Published:** 2019-08-01

**Authors:** Ke Sun, Wei Li, Jun Han, Hong Wu

**Affiliations:** 1 Department of Liver Surgery & Liver Transplantation Center, West China Hospital, Sichuan University, Chengdu, 610041 Sichuan Province China; 2 Department of Critical Care Medicine, Sichuan Provincial Hospital for Women and Children, Chengdu, 610045 Sichuan Province China

**Keywords:** Pancreatic ductal adenocarcinoma, Primary tumor resection, Survival

## Abstract

**Background:**

Previous studies comparing primary tumor resection (PTR) to palliative treatment for advanced-stage pancreatic ductal adenocarcinoma (PDA) were limited by strong selection bias. We used multiple methods to control for confounding and selection bias to estimate the effect of PTR on survival for late-stage PDA.

**Methods:**

Surveillance, Epidemiology, and End Results (SEER) 18 registry database for 2004 through 2014 was retrieved for the present study. A total of 4322 patients with stage III (AJCC, 6th) PDA were included in this study. Propensity score matching (PSM) was performed to eliminate possible bias. In addition, instrumental variable (IV) analysis was utilized to adjust for both measured and unmeasured confounders.

**Results:**

A total of 4322 patients with stage III PDA including 552 (12.8%) who underwent PTR, 3770 (87.2%) without PTR, were identified. In the multivariable cohort, a clear prognostic advantage of PTR was observed in overall survival (OS) (*P* < 0.001) and disease-specific survival (DSS) (P < 0.001) compared to patients after non-surgery therapy. In the PSM cohort, patients in PTR group showed a better OS and DSS (both *P* values < 0.001) compared to patients in non-surgery group. The survival benefit of PTR for stage III PDA was not observed in the two-stage residual inclusion (2SRI) model. Estimates based on this instrument indicated that patients treated with PTR had similar OS (*P* = 0.448) and DSS (*P* = 0.719). In IV analyses stratified by chemotherapy and tumor location, patients undergoing PTR had similar OS and DSS compared to patients in non-surgery group across all subgroups.

**Conclusions:**

Survival with PTR did not differ significantly from palliative treatment in marginal patients with stage III pancreatic adenocarcinoma. High-quality randomized trials are needed to validate these results.

**Electronic supplementary material:**

The online version of this article (10.1186/s12885-019-5966-9) contains supplementary material, which is available to authorized users.

## Background

The incidence of pancreatic ductal adenocarcinoma (PDA) continues to increase. In 2017, an estimated 53670 new cases (female: 25700; male: 27970) of pancreatic cancer were diagnosed within the US and 43090 individuals (female: 20790; male: 22300) were expected to die of the tumor [1]. Primary tumor resection (PTR) is the only curative modality, while more than 80% of tumors were unresectable when present [2]. The 5-year survival of patients with PDA after surgery is approximately 20% (the median survival is 15–23 months) [3–8].

According to American Joint Committee on Cancer (AJCC) classification version 6, patients with stage III PDA (tumors involved celiac axis and/or superior mesenteric artery) can be divided into borderline resectable and unresectable, depending on the extent of the tumor encasement of major vessels [2, 5, 6]. Previously, chemoradiotherapy has been carried out to reduce the risk of a positive surgical margin and distant metastasis [2, 4, 9]. Since the FOLFIRINOX regimen (irinotecan, oxaliplatin, leucovorin, and fluorouracil) was introduced in 2011 by a prospective randomized controlled trial [10], it has been reported to result in objective response rates that were 2–3 fold higher than other regimens in PDA [11]. Several studies have confirmed that a large number of cases even with locally advanced and unresectable PDA can be converted to be resectable by FOLFIRINOX [12–14].

Previous publications have reported a discrepant overall survival of PDA patients with advanced disease undergoing PTR and vascular reconstruction (the median survival ranged from 12 to 35 months) [15–18]. Owing to the varying outcomes regarding to the long-term survival of underline resectable PDA receiving PTR, we designed a population-based cohort study to explore the independent role of PTR in patients with stage III PDA (S-III PDA). We utilized an instrumental variable (IV) analysis to determine variation in outcomes across geographical areas that were different in PTR rates. The IV analysis is aimed to control for potential unknown confounding factors in decision making for surgeries [19, 20]. In the present study, PTR rates in various Health Service Areas (HSA) was employed as our instrument. The treatment option (PTR or non-PTR) for marginal patients (those with a borderline or uncertain need for PTR) may be affected by preferences, beliefs, or surgical skills of surgeons in their HSAs. Patients with S-III PDA would be performed PTR in a high-use HSA, while not in a low-use HSA [21, 22]. The coefficient in the IV analysis represents the adjusted treatment effect for the marginal population rather than the average treatment effect [21].

## Methods

### Patient selection

**Fig. 1:**
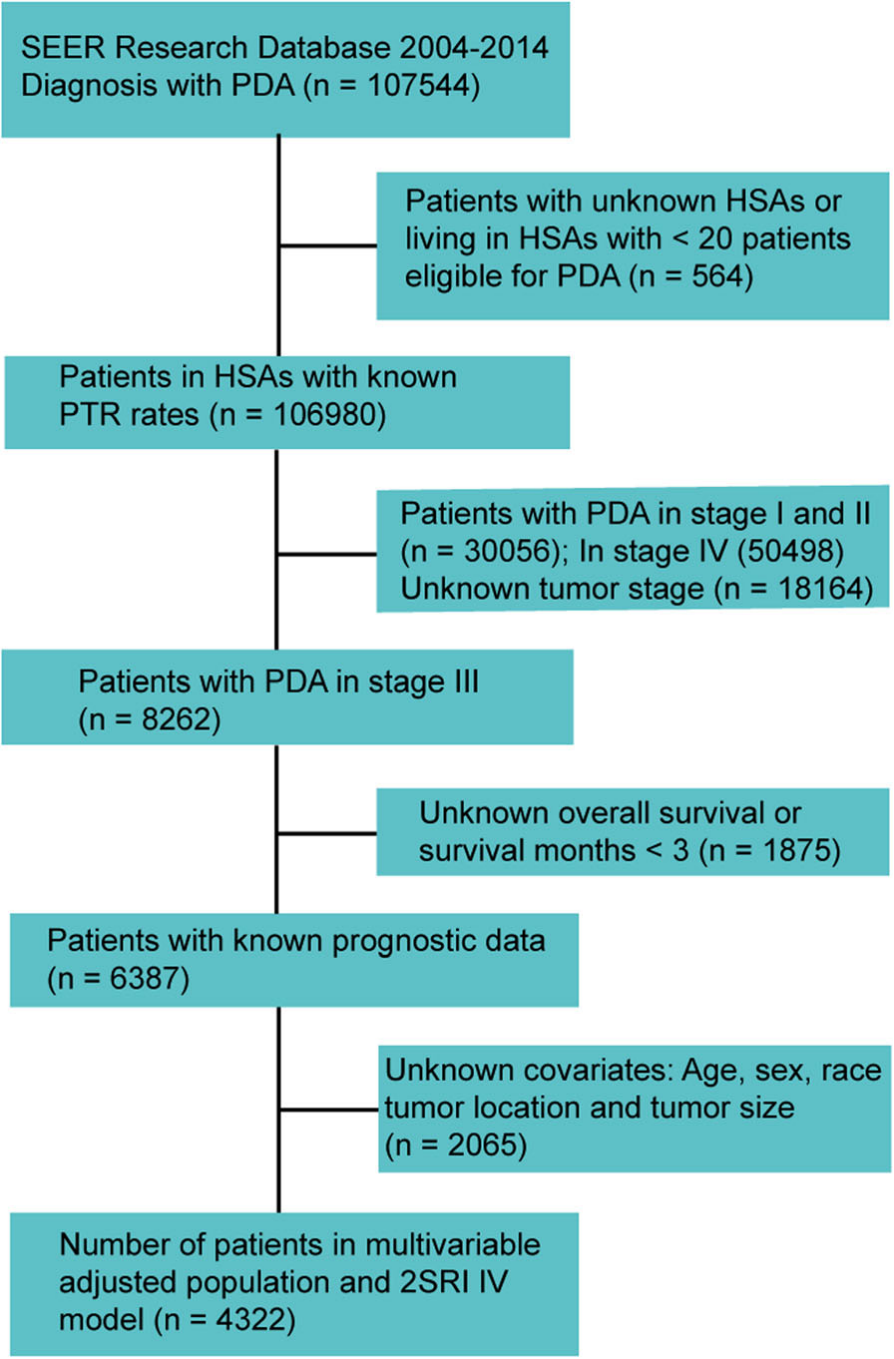
Flowchart representing selection process of patients included in this study

Surveillance, Epidemiology, and End Results (SEER) 18 database for 2004 through 2014 was retrieved for this study (seer.cancer.gov/about/overview.html). The SEER population-based cancer registries covers approximately 28% of the US population, which collects data of tumor incidence, demographics, tumor characteristics and patient survival. Firstly, 107544 patients with PDA was identified based on the pathological diagnosis. The ICD-O-3 (International Classification of Diseases for Oncology, 3rd Edition) site code is C25 and histologic type codes are 8140, 8500, 8010, 8000, 8480, 8481, 8490, 8255, 8021, 8020, 8521, 8141, 8022, 8144. Tumor, node, and metastasis stage of PDA in SEER was based on AJCC stage version 6. The flow diagram of patient selection is shown in Fig. 1. Finally, a total of 4322 cases with stage III PDA were included in the analysis. The following codes related to PDA treatment were selected: PTR: 30 (partial pancreatectomy), 35–37 (Whipple), 40 (whole pancreatectomy), 60 (whole pancreatectomy with subtotal gastrectomy/duodenectomy), 70 (an extended pancreatoduodenectomy) and 80 (pancreatectomy, NOS); none surgical treatment: 0. This study has been approved by the Institutional Review Board at the West China Hospital. All patient data from SEER database is public available and anonymous.

### Statistical analyses

Continuous data are shown as mean ± SD and categorical variables are presented as number (%). The continuous variable was examined by t-test or Kruskal-Wallis H test the categorical data was tested by Chi-square test or Fisher’s exact test. Overall survival (OS) and disease-specific survival (DSS) were the primary endpoints. The former was defined as the time from the date of treatment to the date of death with any cause and the latter was defined as interval until death caused by PDA. The Kaplan-Meier method was used to analyze survival data (compared by the log-rank test). Multivariable analyses were performed by the Cox proportional hazards regressions. Based on previous study [23], the interaction tests were also carried out to identify the interactive factors influencing the relationship between treatment methods and survival.

To further adjust for potential baseline confounding factors, the propensity score matching (PSM) was carried out according to the following parameters: sex, age, race, year of diagnosis, tumor differentiation, tumor size, and chemotherapy. Cases with PTR were matched to those without PTR with a matching ratio of 1:3. The nearest-neighbor PSM was performed by logistic regression.

PTR rates in HSAs were utilized as an IV. In this study, the PTR rate in HSAs is a qualified instrument because it is highly associated with the probability of a patient’s exposure to primary tumor resection (F statistic > 10) and is also not related to patient survival. In addition, covariate balance across quintiles was also examined. We did not include patients living in HSAs with fewer than 20 patients, given the difficulty to confirm the PTR rates in those HSAs [24]. The two-stage residual inclusion (2SRI) method was utilized for instrumental variable analyses [25].

## Results

### Patient demographics

A total of 4322 S-III PDA patients including 552 (12.8%) who underwent PTR, 3770 (87.2%) without PTR, were identified. Table 1 displayed the general demographics of the final cohort of 4322 S-III PDA patients with available variables. The mean age of patients undergoing PTR and none was 64.6 and 66.7 years, respectively. Compared with the non-surgery group, the PTR group had more pancreatic head tumor (79.3% vs. 70.9%) and smaller tumor size (38.5 ± 16.3 mm vs. 41.0 ± 15.4 mm). The other clinicopathologic characteristics including sex, race and number of patients receiving chemotherapy showed no statistically significance between PTR and non-surgery groups (all *P* > 0.05).

**Table 1:** Clinical features of the included patients with PDA

Variable	Before PSM			After PSM		
	None (*n* = 3770)	PTR (*n* = 552)	*P* value	None (*n* = 720)	PTR (*n* = 240)	*P* value
Age (years)	66.7 ± 11.3	64.6 ± 10.5	< 0.001	67.4 ± 11.1	67.2 ± 10.2	0.777
Sex			0.510			0.911
Female	1887 (50.1%)	268 (48.6%)		361 (50.1%)	122 (50.8%)	
Male	1883 (49.9%)	284 (51.4%)		359 (49.9%)	118 (49.2%)	
Race			0.528			0.161
White	2968 (78.7%)	445 (80.6%)		563 (78.2%)	197 (82.1%)	
Black	463 (12.3%)	59 (10.7%)		93 (12.9%)	20 (8.3%)	
Other	339 (9.0%)	48 (8.7%)		64 (8.9%)	23 (9.6%)	
Primary tumor site in pancreas			< 0.001			0.463
Head	2672 (70.9%)	438 (79.3%)		501 (69.6%)	173 (72.1%)	
Body and tail	1080 (28.6%)	116 (19.2%)		219 (30.4%)	67 (27.9%)	
Tumor size (mm)	41.0 ± 15.4	38.5 ± 16.3	< 0.001	44.9 ± 16.7	45.6 ± 18.6	0.558
Tumor differentiation			< 0.001			0.197
I	178 (4.7%)	52 (9.4%)		57 (7.9%)	17 (7.1%)	
II	402 (10.7%)	223 (40.4%)		153 (21.2%)	56 (23.3%)	
III	421 (11.2%)	163 (29.5%)		168 (23.3%)	53 (22.1%)	
IV	26 (0.7%)	3 (0.5%)		1 (0.1%)	3 (1.2%)	
Chemotherapy			0.272			0.228
No/unknown	2960 (78.5%)	422 (76.4%)		608 (84.4%)	194 (80.8%)	
Yes	810 (21.5%)	130 (23.6%)		112 (15.6%)	46 (19.2%)	

Data are shown as mean ± SD or n (%). PTR, primary tumor resection. Tumor differentiation: I, well-differentiated; II, moderate- differentiated; III, poor-differentiated; IV, un-differentiated

### Multivariable analyses

**Fig. 2:**
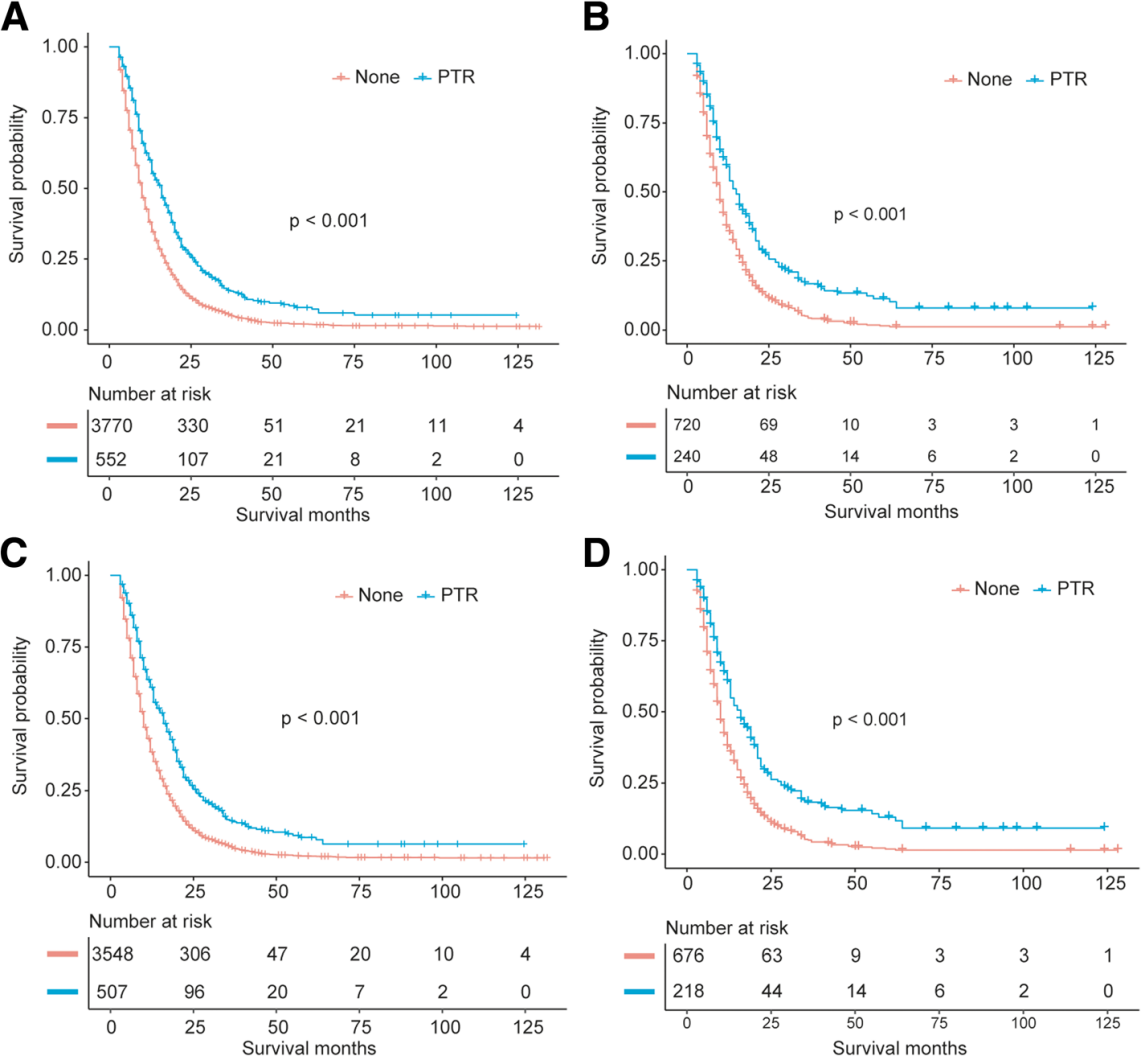
**a**: Overall survival analysis for patients undergoing PTR and none in non-adjusted population. **b**: Overall survival analysis for patients after PTR and none in propensity score matched population. **c**: Disease-specific survival analysis for patients undergoing PTR and none in non-adjusted population. **d**: Disease-specific survival analysis for patients after PTR and none in propensity score matched population

In multivariable analyses, we included a total of 4322 patients with known prognostic data. The mean overall survival time (in the total cohort) for patients who underwent PTR and patients undergoing non-surgery were 23.9 months and 14.5 months, respectively. The mean DSS time for cases after PTR and none were 23.9 and 15.4 months, respectively. Patients with PTR had longer OS (*P* < 0.001) and DSS (*P* < 0.001) compared to patients with non-surgery treatment (Fig. 2a and c).

In the cohort for multivariable analyses (OS: *n* = 4322; DSS: *n* = 4055), after adjusting for potential confounding factors, a clear prognostic advantage of PTR was observed in OS (HR, 0.59; 95% CI, 0.53 to 0.66; *P* < 0.001) and DSS (HR, 0.57; 95% CI, 0.51 to 0.65; *P* < 0.001) compared to patients after non-surgery therapy (Table 2).

**Table 2:** Association of PTR with patient overall survival in locally advanced PDA patients

	OS			DSS		
	Number	HR (95%CI)	*P* value	Number	HR (95% CI)	*P* value
Non-adjusted	4322	0.61 (0.55, 0.68)	< 0.001	4055	0.60 (0.54, 0.66)	< 0.001
Multivariable adjusted model*	4322	0.59 (0.53, 0.66)	< 0.001	4055	0.57 (0.51, 0.65)	< 0.001
Matched on propensity score	960	0.59 (0.50, 0.69)	< 0.001	960	0.57 (0.47, 0.67)	< 0.001
Regression adjusted with propensity score						
Propensity score, continuous	4322	0.61 (0.55, 0.67)	< 0.001	4055	0.61 (0.54, 0.68)	< 0.001
Propensity score, quintile	4322	0.62 (0.56, 0.69)	< 0.001	4055	0.60 (0.53, 0.67)	< 0.001

PTR was the reference (HR: 1). Data are shown as HR (95% CI) *P* value. *Adjusted model was adjusted for: age, race, sex, year of diagnosis, tumor differentiation, tumor size, tumor location and chemotherapy

### Instrumental variable analysis

To be valid, an instrumental variable must meet two conditions: 1) the variable must be highly associated with the treatment of interest (in this study receipt of PTR); and 2) the instrumental variable cannot be related to the outcomes (in this study survival) except through its effect on the treatment received [26].

The average PTR rate in HSAs fluctuated from a low of 16% (quintile 1) to a high of 27% (quintile 5). The F-statistics was 3325.8 (*P* < 0.001), indicating that the instrument was strongly related to the treatment. In addition, in a standard COX regression, no significant correlation was observed between the IV and OS (HR 1.18, 95% CI 0.27–5.05, *P* = 0.827). We divided patients into quintiles according to the proportion of cases within each HSA who underwent PTR (Additional file 1: Table S1). Most of the clinicopathologic features were balanced across quintiles. Consequently, these observations suggest that HSA PTR rate meets the two requirements for a valid instrument.

For patients with S-III PDA, the salutary benefit of PTR in survival was not observed in the 2SRI model. In IV analysis, results indicated that patients who underwent PTR had similar OS (HR 0.74, 95% CI 0.34–1.61, *P* = 0.448) and DSS (HR 0.86, 95% CI 0.38–1.94, *P* = 0.719) after adjusting confounding factors (Table 3).

**Table 3:** Instrumental variable analysis of the impact of PTR on survival for patients with stage III locally advanced PDA in 2SRI IV model

	OS (*n* = 4322)			DSS (*n* = 4055)		
	HR	95% CI	*P*-value	HR	95% CI	*P*-value
PTR vs. none	0.741	0.342, 1.607	0.448	0.862	0.384–1.935	0.719
Age, years	1.013	1.010–1.017	< 0.001	1.015	1.011–1.018	< 0.001
Sex, male vs. female	1.112	1.043–1.187	0.001	1.104	1.032–1.180	0.004
Race						
Black vs. White	1.080	0.974–1.197	0.146	1.085	0.974–1.209	0.137
Other vs. White	0.978	0.872–1.097	0.700	0.990	0.878–1.116	0.871
Primary tumor site in pancreas						
Body vs. head	0.955	0.874–1.045	0.317	0.954	0.870–1.046	0.314
Tail vs. head	1.051	0.886–1.246	0.570	0.994	0.829–1.191	0.948
Tumor size, cm	1.003	1.001–1.005	< 0.001	1.004	1.002–1.007	< 0.001
Tumor differentiation						
II vs. I	1.157	0.981–1.366	0.084	1.196	1.005–1.424	0.044
III vs. I	1.575	1.334–1.860	< 0.001	1.628	1.368–1.938	< 0.001
IV vs. I	1.806	1.181–2.762	0.006	1.811	1.171–2.799	0.008
Year of diagnosis, 2010–2014 vs. 2004–2009	0.987	0.981–0.994	< 0.001	0.984	0.977–0.990	< 0.001

OS, overall survival; DSS, disease-specific survival; HR, hazard ratios; CI, confidence interval. Tumor differentiation: I, well-differentiated; II, moderate- differentiated; III, poor-differentiated; IV, un-differentiated

### Subgroup analyses

In IV analyses stratified by chemotherapy, we found that the similar effects of PTR vs. none on patient survival were consistent across both subgroups (Table 4). In IV analyses, patients in the PTR group receiving chemotherapy had similar OS (HR 0.43, 95% CI 0.09–2.15, *P* = 0.304) and DSS (HR 0.56, 95% CI 0.10–3.14, *P* = 0.508) compared to patients in the non-PTR group receiving chemotherapy. In IV analyses stratified by tumor location, we found that the similar effects of PTR vs. non-PTR on survival (both OS and DSS) were consistent across all subgroups with different tumor location (Table 5).

**Table 4:** Subgroup analyses according to chemotherapy (P for interaction: 0.679)

	No chemotherapy or unknown	Chemotherapy
OS		
Non-adjusted	0.58 (0.51, 0.65) < 0.001	0.67 (0.55, 0.81) < 0.001
Adjusted		
Traditional regression model	0.58 (0.51, 0.66) < 0.001	0.61 (0.48, 0.76) < 0.001
2SRI IV model	1.27 (0.51, 3.13) 0.610	0.43 (0.09, 2.15) 0.304
DSS		
Non-adjusted	0.55 (0.49, 0.63) < 0.001	0.67 (0.55, 0.82) < 0.001
Adjusted		
Traditional regression model	0.56 (0.49, 0.64) < 0.001	0.61 (0.48, 0.77) < 0.001
2SRI IV model	1.31 (0.51, 3.34) 0.573	0.56 (0.10, 3.14) 0.508

PTR was the reference (HR: 1). Data are shown as HR (95%CI) *P* value. All adjusted models were adjusted for: age, race, sex, year of diagnosis, tumor location and tumor size. OS, overall survival; DSS, disease-specific survival; 2SRI, 2 stage residual inclusion; IV, instrumental variable

**Table 5:** Subgroup analyses according to tumor location

	Pancreatic head	Pancreatic body	Pancreatic tail
OS			
Non-adjusted	0.60 (0.54, 0.67) < 0.001	0.48 (0.35, 0.66) < 0.001	0.81 (0.57, 1.17) 0.264
Adjusted			
Traditional regression model	0.58 (0.51, 0.66) < 0.001	0.55 (0.39, 0.76) < 0.001	0.54 (0.35, 0.86) 0.009
2SRI IV model	0.43 (0.12, 1.48) 0.181	0.39 (0.01, 18.80) 0.637	1.57 (0.01, 217.96) 0.858
DSS			
Non-adjusted	0.58 (0.52, 0.66) < 0.001	0.48 (0.34, 0.66) < 0.001	0.85 (0.58, 1.24) 0.392
Adjusted			
Traditional regression model	0.57 (0.50, 0.64) < 0.001	0.53 (0.37, 0.75) < 0.001	0.53 (0.32, 0.87) 0.013
2SRI IV model	0.72 (0.20, 2.62) 0.614	0.44 (0.01, 24.44) 0.689	0.90 (0.00, 203.30) 0.970

PTR was the reference (HR: 1). Data are shown as HR (95%CI) *P* value. All adjusted models were adjusted for: age, race, sex, year of diagnosis, tumor size and chemotherapy. OS, overall survival; DSS, disease-specific survival; 2SRI, 2 stage residual inclusion; IV, instrumental variable

In IV analyses stratified by the other clinicopathologic characteristics (sex, race, age, year of diagnosis and tumor size.), we found that the treatment effect of PTR (OS and DSS) was consistent across all the subgroups (data not shown).

### Propensity score matched analyses

In the propensity-matched population, all the potential prognosis-relevant characteristics were well-balanced for most of the baseline features (Table 1). In the PSM cohort, results from the univariate analysis indicated that cases with PTR had better OS (HR 0.59, 95% CI 0.50–0.69 *P* < 0.001) and DSS (HR 0.57, 95% CI 0.47–0.67, *P* < 0.001) compared to patients with non-surgery treatment (Table 2). In the PSM-adjusted population, patients in PTR group still showed a better OS and DSS (both *P* values < 0.001) compared to patients in non-surgery group by Kaplan-Meier method (Fig. 2b and d).

The HRs (PTR vs. none) adjusted by propensity score showed both longer OS (continuous: HR 0.61, 95% CI 0.55–0.67, *P* < 0.001; quintile: HR 0.62, 95% CI 0.56–0.69, *P* < 0.001) and DSS (continuous: HR 0.61, 95% CI 0.54–0.68, *P* < 0.001; quintile: HR 0.60, 95% CI 0.53–0.67, *P* < 0.001) associated with PTR (Table 2).

## Discussion

Pancreatic resection is associated with better outcomes for early-stage PDA. [27] However, for patients with underline resectable PDA, though chemotherapy regimen such as FOLFIRINOX increased the tumor resectability, the long-term survival (OS and DSS) in this study was comparable to those receiving non-surgery treatment in IV analyses. This conclusion is inconsistent with previous studies demonstrating that S-III PDA patients had a better survival after PTR compared to those without surgery [15, 18, 28–30].

Among previous studies related to PTR versus non-surgical management in cases with advanced PDA, there were two randomized controlled trials (RCTs) [28, 29] comparing PTR versus non-surgical treatments in cases with underline resectable PDA. Both RCTs included patients with locally advanced PDA invading the serosa anteriorly or retroperitoneum posteriorly or involving the major vascular structures. One RCT enrolled cases with PDA in different location of the pancreas and another included only cases with tumor in the pancreatic head or neck. Both studies demonstrated that patients receiving PTR and vascular resection and reconstruction had longer survival compared to patients only undergoing non-surgery treatment such as chemoradiotherapy. However, both researches were at high risk of bias and only a small number of patients (47 and 51 patients in two studies, respectively) were included.

In this study, utilizing IV analyses, we concluded that patients with S-III PDA receiving PTR had a similar long-term prognosis compared to those without PTR (only receive non-surgery treatments). We have applied both traditional regression analyses and propensity score methods to explore relations between surgical methods and long-term survival. However, these analytic methods cannot adjust for unknown confounding factors [15, 26]. In contrast, results from IV analyses (2SRI model) were observed to be more close to outcomes from RCTs [26]. Given the lack of high-quality RCTs associated with PTR vs. none for PDA patients, results of IV analyses may represent the best evidence available to guide treatment decision-making. It should be noted that IV analyses estimate the treatment effect on the marginal population rather than the average treatment effect of PTR [19, 21]. The marginal population represents the population that would receive PTR in a high-use HSA but not in a low-use HSA. The IV analysis does not rely on defining the specific clinical parameters of these populations. Instead, it is based on the hypothesis that patients reside randomly around hospitals and some patients are treated differently in different centers.

**Fig. 3:**
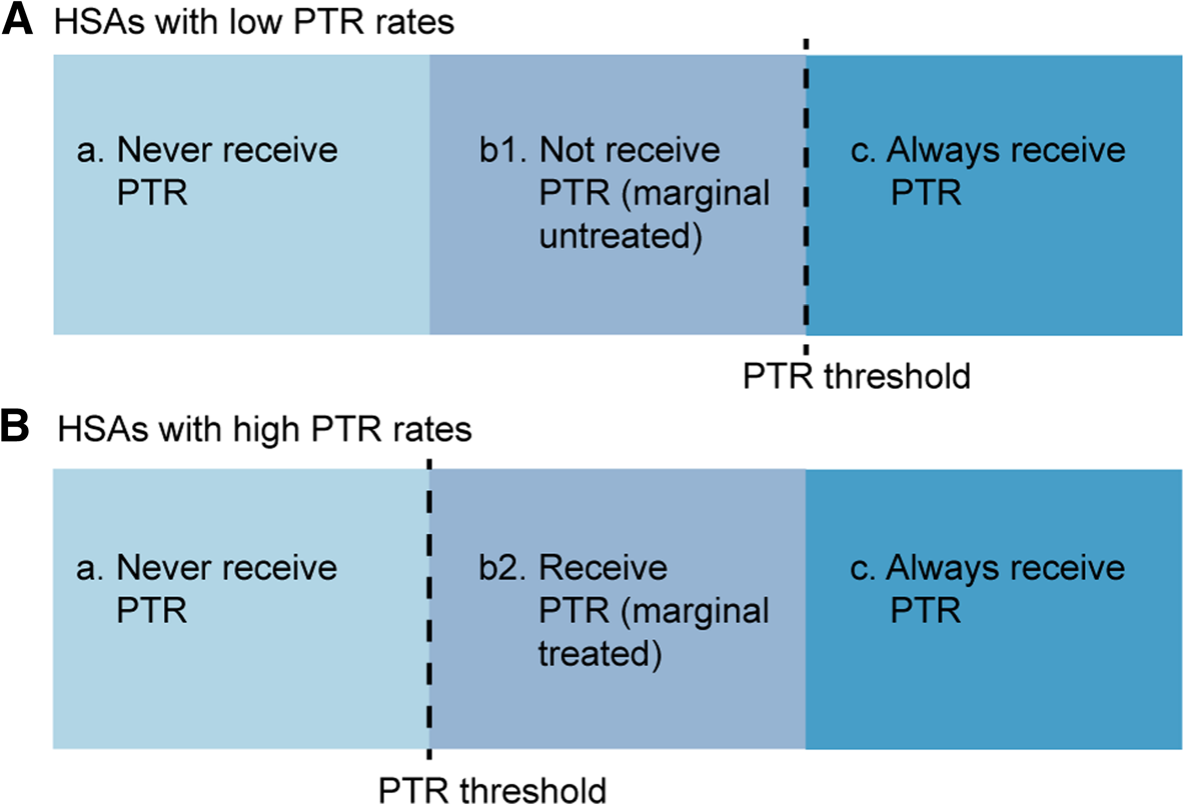
In **a**, the hospital performs PTR less and has a higher threshold for PTR while in **b**, the hospital performs PTR more frequently and has a lower threshold for carrying out PTR. In both hospitals, there will be a population of patients who would never be performed PTR (group “a”) as well as patients who would always be performed PTR (group “c”). At the same time, there will be a group of patients who would either not be performed (group “b1”) or would be performed (group “b2”) PTR solely because of the hospital to which they presented. These “b” groups together are the marginal population

There are several limitations to this study. First, although we have acquired the data related to the chemotherapy from SEER database, the detailed regimens and the timing of chemotherapy were yet inaccessible. Patients without chemotherapy or with unknown data of chemotherapy accounted for nearly 80% of patients with pancreatic cancer. In addition, we cannot divide S-III PDA into borderline resectable and unresectable groups based on the extension of tumor invasion, thus we cannot assess whether patients with surgical resection was well chosen. The proportion of patients with R0 tumor resection could not be confirmed in this study. Second, patient performance status and presence of comorbidities are risk factors for patient prognosis. However, the SEER database does not provide these data, thus we cannot adjust these factors by multivariable analyses. Third, details on postoperative morbidities were extremely limited, thus we could not evaluate the influence of treatment methods to the short-term prognosis. Fourth, the observations of this study should be interpreted cautiously, due to some patients with unknown covariates in the SEER database were excluded from the analyses. Finally, even though treatment rate (PTR rate) is a useful practical IV, there remains potential for instrument-outcome confounding, such as receipt of other treatments also associated with our instrument and the outcome. In addition, IV analysis only estimates the effect on marginal population, while the marginal population excludes patients who would always or never receive PTR, focusing on PDA patients whose indications for PTR are more uncertain (Fig. 3). [20, 26, 31]

## Conclusions

In conclusion, by integrating results from multivariate COX regression, PSM models and IV analysis, our study demonstrated that PTR provided similar overall and tumor-specific survival benefits in cases with stage III PDA compared to patients with palliative treatments. Further high-quality prospective randomized trials are needed to validate this conclusion and further investigations are required to identify late-stage patients suitable for PTR.

## Additional file

Additional file 1: **Table S1.** Characteristics of patients by quintile of Health Services Area PTR rates. (DOCX 18 kb)

## Data Availability

All primary data is available by sending email to: 13881958922@163.com or downloading from SEER database.

## Abbreviations

AJCC: American Joint Committee on Cancer
CI: Confidence interval
DSS: Disease-specific survival
HR: Hazard ratio
NCCN: National Comprehensive Cancer Network
OS: Overall survival
PDA: Pancreatic adenocarcinoma
PSM: Propensity score matching
PTR: Primary tumor resection
SEER: Surveillance, Epidemiology, and End Results

## References

[1] Siegel RL, Miller KD, Jemal A (2017). Cancer statistics, 2017. CA Cancer J Clin.

[2] Tempero MA, Malafa MP, Al-Hawary M, Asbun H, Bain A, Behrman SW, Benson AB, Binder E, Cardin DB, Cha C (2017). Pancreatic adenocarcinoma, version 2.2017, NCCN clinical practice guidelines in oncology. J Natl Compr Canc Netw.

[3] Yeo CJ, Abrams RA, Grochow LB, Sohn TA, Ord SE, Hruban RH, Zahurak ML, Dooley WC, Coleman J, Sauter PK (1997). Pancreaticoduodenectomy for pancreatic adenocarcinoma: postoperative adjuvant chemoradiation improves survival. A prospective, single-institution experience. Ann Surg.

[4] Neoptolemos JP, Stocken DD, Bassi C, Ghaneh P, Cunningham D, Goldstein D, Padbury R, Moore MJ, Gallinger S, Mariette C (2010). Adjuvant chemotherapy with fluorouracil plus folinic acid vs gemcitabine following pancreatic cancer resection: a randomized controlled trial. Jama.

[5] Sabater L, Munoz E, Rosello S, Dorcaratto D, Garces-Albir M, Huerta M, Roda D, Gomez-Mateo MC, Ferrandez-Izquierdo A, Darder A (2018). Borderline resectable pancreatic cancer. Challenges and controversies. Cancer Treat Rev.

[6] Lopez NE, Prendergast C, Lowy AM (2014). Borderline resectable pancreatic cancer: definitions and management. World J Gastroenterol.

[7] Varadhachary GR, Tamm EP, Abbruzzese JL, Xiong HQ, Crane CH, Wang H, Lee JE, Pisters PW, Evans DB, Wolff RA (2006). Borderline resectable pancreatic cancer: definitions, management, and role of preoperative therapy. Ann Surg Oncol.

[8] Springett GM, Hoffe SE (2008). Borderline resectable pancreatic cancer: on the edge of survival. Cancer control : journal of the Moffitt Cancer Center.

[9] Chin V, Nagrial A, Sjoquist K, O'Connor CA, Chantrill L, Biankin AV, Scholten RJ, Yip D (2018). Chemotherapy and radiotherapy for advanced pancreatic cancer. Cochrane Database Syst Rev.

[10] Vaccaro V, Sperduti I, Milella M (2011). FOLFIRINOX versus gemcitabine for metastatic pancreatic cancer. N Engl J Med.

[11] Sadot E, Doussot A, O'Reilly EM, Lowery MA, Goodman KA, Do RK, Tang LH, Gonen M, D'Angelica MI, DeMatteo RP (2015). FOLFIRINOX induction therapy for stage 3 pancreatic adenocarcinoma. Ann Surg Oncol.

[12] Luu AM, Herzog T, Hoehn P, Reinacher-Schick A, Munding J, Uhl W, Braumann C (2018). FOLFIRINOX treatment leading to pathologic complete response of a locally advanced pancreatic cancer. J Gastrointest Oncol.

[13] Nitsche U, Wenzel P, Siveke JT, Braren R, Holzapfel K, Schlitter AM, Stoss C, Kong B, Esposito I, Erkan M (2015). Resectability after first-line FOLFIRINOX in initially Unresectable locally advanced pancreatic Cancer: a single-center experience. Ann Surg Oncol.

[14] Ferrone CR, Marchegiani G, Hong TS, Ryan DP, Deshpande V, McDonnell EI, Sabbatino F, Santos DD, Allen JN, Blaszkowsky LS (2015). Radiological and surgical implications of neoadjuvant treatment with FOLFIRINOX for locally advanced and borderline resectable pancreatic cancer. Ann Surg.

[15] Wang L, Cheng CS, Chen L, Chen Z (2018). Benefit from the inclusion of surgery in the treatment of patients with stage III pancreatic cancer: a propensity-adjusted, population-based SEER analysis. Cancer Manag Res.

[16] Beane JD, House MG, Pitt SC, Kilbane EM, Hall BL, Parmar AD, Riall TS, Pitt HA (2015). Distal pancreatectomy with celiac axis resection: what are the added risks?. HPB (Oxford)..

[17] Denecke T, Andreou A, Podrabsky P, Grieser C, Warnick P, Bahra M, Klein F, Hamm B, Neuhaus P, Glanemann M (2011). Distal pancreatectomy with en bloc resection of the celiac trunk for extended pancreatic tumor disease: an interdisciplinary approach. Cardiovasc Intervent Radiol.

[18] Klompmaker S, de Rooij T, Korteweg JJ, van Dieren S, van Lienden KP, van Gulik TM, Busch OR, Besselink MG (2016). Systematic review of outcomes after distal pancreatectomy with coeliac axis resection for locally advanced pancreatic cancer. Br J Surg.

[19] McDowell BD, Chapman CG, Smith BJ, Button AM, Chrischilles EA, Mezhir JJ (2015). Pancreatectomy predicts improved survival for pancreatic adenocarcinoma: results of an instrumental variable analysis. Ann Surg.

[20] Baiocchi M, Cheng J, Small DS (2014). Instrumental variable methods for causal inference. Stat Med.

[21] Valley TS, Sjoding MW, Ryan AM, Iwashyna TJ, Cooke CR (2015). Association of Intensive Care Unit Admission with Mortality among Older Patients with Pneumonia. Jama.

[22] Tan HJ, Norton EC, Ye Z, Hafez KS, Gore JL, Miller DC (2012). Long-term survival following partial vs radical nephrectomy among older patients with early-stage kidney cancer. Jama.

[23] Zhao J, Mao J, Li W (2019). Association of Tumor Grade with Long-Term Survival in patients with hepatocellular carcinoma after liver transplantation. Transplant Proc.

[24] Xu H, Xia Z, Jia X, Chen K, Li D, Dai Y, Tao M, Mao Y (2015). Primary tumor resection is associated with improved survival in stage IV colorectal Cancer: an instrumental variable analysis. Sci Rep.

[25] Gore JL, Litwin MS, Lai J, Yano EM, Madison R, Setodji C, Adams JL, Saigal CS (2010). Use of radical cystectomy for patients with invasive bladder cancer. J Natl Cancer Inst.

[26] Terza JV, Basu A, Rathouz PJ (2008). Two-stage residual inclusion estimation: addressing endogeneity in health econometric modeling. J Health Econ.

[27] Mohammed S, Van Buren G, Fisher WE (2014). Pancreatic cancer: advances in treatment. World J Gastroenterol.

[28] Doi R, Imamura M, Hosotani R, Imaizumi T, Hatori T, Takasaki K, Funakoshi A, Wakasugi H, Asano T, Hishinuma S (2008). Surgery versus radiochemotherapy for resectable locally invasive pancreatic cancer: final results of a randomized multi-institutional trial. Surg Today.

[29] Lygidakis NJ, Singh G, Bardaxoglou E, Dedemadi G, Sgourakis G, Nestoridis J, Malliotakis A, Pedonomou M, Solomou EK, Safioleas M (2004). Mono-bloc total spleno-pancreaticoduodenectomy for pancreatic head carcinoma with portal-mesenteric venous invasion. A prospective randomized study. Hepato-gastroenterology.

[30] Scemama U, Birnbaum DJ, Ouaissi M, Turrini O, Moutardier V, Soussan J (2016). Portal vein stent placement in five patients with chronic portal vein thrombosis prior to pancreatic surgery. J Vasc Interv Radiol.

[31] Wan Fei, Small Dylan, Bekelman Justin E., Mitra Nandita (2015). Bias in estimating the causal hazard ratio when using two-stage instrumental variable methods. Statistics in Medicine.

