# Supplementary material for: Comparative effectiveness of primary tumor resection in patients with stage III pancreatic adenocarcinoma
Source: BMC Cancer. 2019 Aug 1;19:761. doi: 10.1186/s12885-019-5966-9 (PMC6676580; doi:10.1186/s12885-019-5966-9)
Supplement: Supplementary file 1 — Table S1. Characteristics of patients by quintile of Health Services Area PTR rates. (DOCX 18 kb) [file 12885_2019_5966_MOESM1_ESM.docx]

| Table S1. Characteristics of patients by quintile of Health Services Area PTR rates. | | | | | |
| --- | --- | --- | --- | --- | --- |
| Variable | Area resection rate quintile | | | | |
|  | Quintile 1 (n=781) | Quintile 2 (n=772) | Quintile 3 (n=915) | Quintile 4 (n=893) | Quintile 5 (n=961) |
| PTR rates in HSAs | 0.16 ± 0.01 | 0.19 ± 0.01 | 0.21 ± 0.01 | 0.23 ± 0.01 | 0.27 ± 0.02 |
| Age (years) | 66.4 ± 10.5 | 66.6 ± 11.5 | 66.2 ± 11.4 | 66.2 ± 11.3 | 66.6 ± 11.2 |
| Sex |  |  |  |  |  |
| Female | 360 (46.1%) | 388 (50.3%) | 474 (51.8%) | 449 (50.3%) | 484 (50.4%) |
| Male | 421 (53.9%) | 384 (49.7%) | 441 (48.2%) | 444 (49.7%) | 477 (49.6%) |
| Race |  |  |  |  |  |
| White | 658 (84.3%) | 619 (80.2%) | 685 (74.9%) | 692 (77.5%) | 759 (79.0%) |
| Black | 52 (6.7%) | 96 (12.4%) | 139 (15.2%) | 154 (17.2%) | 81 (8.4%) |
| Other | 71 (9.1%) | 57 (7.4%) | 91 (9.9%) | 47 (5.3%) | 121 (12.6%) |
| Primary tumor site in pancreas |  |  |  |  |  |
| Body | 572 (73.2%) | 552 (71.5%) | 659 (72.0%) | 632 (70.8%) | 695 (72.3%) |
| Head | 176 (22.5%) | 179 (23.2%) | 206 (22.5%) | 214 (24.0%) | 212 (22.1%) |
| Tail | 30 (3.8%) | 38 (4.9%) | 39 (4.3%) | 42 (4.7%) | 50 (5.2%) |
| Tumor size (mm) | 41.8 ± 15.7 | 40.4 ± 15.0 | 41.4 ± 15.3 | 40.3 ± 15.7 | 39.7 ± 16.1 |
| Tumor differentiation |  |  |  |  |  |
| I | 38 (4.9%) | 39 (5.1%) | 49 (5.4%) | 55 (6.2%) | 49 (5.1%) |
| II | 120 (15.4%) | 99 (12.8%) | 135 (14.8%) | 122 (13.7%) | 149 (15.5%) |
| III | 103 (13.2%) | 120 (15.5%) | 116 (12.7%) | 113 (12.7%) | 132 (13.7%) |
| IV | 6 (0.8%) | 3 (0.4%) | 9 (1.0%) | 3 (0.3%) | 8 (0.8%) |
| Chemotherapy |  |  |  |  |  |
| No/unknown | 611 (78.2%) | 575 (74.5%) | 722 (78.9%) | 711 (79.6%) | 763 (79.4%) |
| Yes | 170 (21.8%) | 197 (25.5%) | 193 (21.1%) | 182 (20.4%) | 198 (20.6%) |
| PTR, primary tumor resection; HSA, health service area. Tumor differentiation: I, well-differentiated; II, moderate- differentiated; III, poor-differentiated; IV, un-differentiated. | | | | | |
